# Lung immune challenge study protocol: controlled exposure to inhaled resiquimod (R848) to study mechanisms of inflammation

**DOI:** 10.1093/immadv/ltaf005

**Published:** 2025-02-24

**Authors:** Akhilesh Jha, Marie Fisk, Jamie Forrester, Jacqui Galloway, Jade Joseph, Robyn Staples, Karl P Sylvester

**Affiliations:** Victor Phillip Dahdaleh Heart & Lung Research Institute, Department of Medicine, University of Cambridge, Cambridge, England; Department of Respiratory Medicine, Cambridge University Hospitals NHS Foundation Trust, Cambridge, England; Victor Phillip Dahdaleh Heart & Lung Research Institute, Department of Medicine, University of Cambridge, Cambridge, England; Department of Respiratory Medicine, Cambridge University Hospitals NHS Foundation Trust, Cambridge, England; Department of Respiratory Medicine, Cambridge University Hospitals NHS Foundation Trust, Cambridge, England; Department of Respiratory Medicine, Cambridge University Hospitals NHS Foundation Trust, Cambridge, England; Victor Phillip Dahdaleh Heart & Lung Research Institute, Department of Medicine, University of Cambridge, Cambridge, England; Department of Pharmacy, Cambridge University Hospitals NHS Foundation Trust, Cambridge, England; Cambridge Respiratory Physiology, Royal Papworth & Cambridge University Hospitals NHS Foundation Trusts, Cambridge, England

**Keywords:** respiratory medicine, immunology, innate immunity, mucosal immunology, asthma, human challenge

## Abstract

This study aims to develop a human lung immune challenge model using inhaled Resiquimod (R848), a Toll-like receptor 7/8 agonist, to investigate inflammatory mechanisms involved in the human respiratory mucosa in health and disease. This approach seeks to induce innate immune anti-viral responses in the lungs and blood, with a suitable dose of inhaled R848 that is clinically tolerable. The study will include healthy volunteers and individuals with asthma. The primary outcome is a change in CXCL10, a biomarker representative of anti-viral responses, at 24 hours post-exposure. Secondary outcomes include changes in lung function, physiological parameters, and inflammatory markers, including C-reactive protein and eosinophil counts. This trial involves a single ascending dose, randomized, single-blind, placebo-controlled design. Participants will receive R848 via nebulization in escalating doses from 0.1 to 100 µg/ml or saline placebo. Safety assessments include spirometry, vital signs, and blood samples to monitor systemic and lung-specific immune responses. The study will contribute to understanding immune pathways in asthma and provide a platform for testing novel anti-inflammatory therapeutics. The protocol has been approved by relevant ethics committees and will be disseminated via peer-reviewed publications and open-access data repositories.

## Graphical Abstract

**Graphical Abstract:**
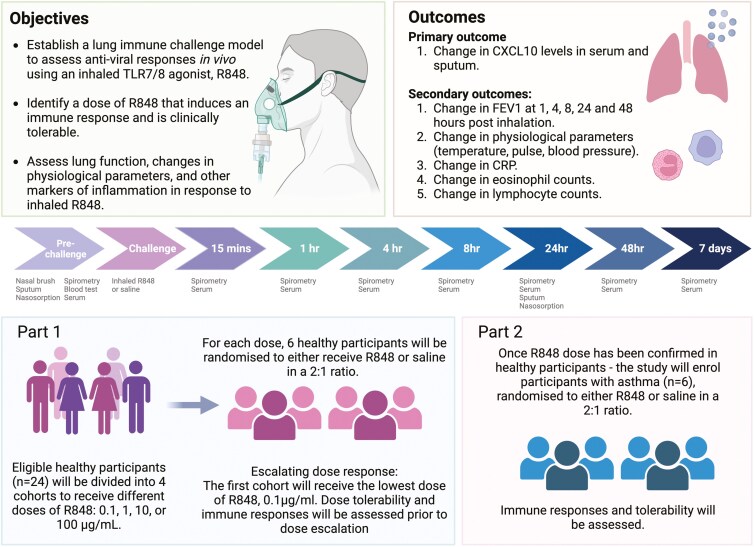

## Introduction

### Background

Acute respiratory viral infections represent a major cause of morbidity, particularly in individuals with underlying respiratory conditions such as asthma and chronic obstructive pulmonary disease. These infections are frequently implicated in asthma exacerbations, a condition associated with inflammation of the airways. Although biologic therapies have been developed to target chronic allergic inflammation in asthma, acute asthma attacks continue to be managed with corticosteroids, which have broad immunosuppressive effects and variable efficacy. There remains a need for more specific treatments that modulate the underlying immune response during exacerbations [1].

Toll-like receptors (TLRs) are pivotal in detecting viral pathogens and initiating innate immune responses, which are crucial for the body’s defence against respiratory viruses [2]. TLR7 and TLR8 are intracellular pattern recognition receptors that recognize single-stranded RNA viruses, such as influenza and SARS-CoV-2. The TLR7/8 agonist Resiquimod (R848) stimulates antiviral responses by activating interferon production, inflammatory cytokines, and chemokines, such as CXCL10, which help recruit immune cells to the site of infection [3–5].

## Rationale

In previous human studies, intranasal administration of R848 has been shown to induce local immune responses in healthy volunteers and individuals with asthma and allergic rhinitis [6, 7]. This approach offers a valuable tool for studying antiviral immunity and inflammation without the risks associated with live viral challenges. However, the nasal mucosa differs from the lung environment, particularly in patients with asthma, where structural changes such as airway remodelling may influence immune responses [8, 9]. Thus, an approach specifically investigating pulmonary anti-viral responses is crucial for gaining insights into the immune mechanisms driving asthma exacerbations and other inflammatory respiratory diseases. The objectives of the trial will mirror other approaches used to study lung immune responses such as studies that utilize inhaled lipopolysaccharide to mimic bacterial infections [10].

## Objectives

This study’s objective is to establish a lung immune challenge model using inhaled R848 to induce a controlled, measurable immune response in the lungs and blood. The primary objective is to identify a dose of R848 that induces measurable changes in CXCL10, a biomarker of antiviral immune activation and is clinically tolerable. Secondary objectives include assessing lung function, changes in physiological parameters, and other markers of inflammation in response to inhaled R848. This model will provide a platform for future studies aimed at testing novel therapeutic agents targeting viral-induced asthma exacerbations and other respiratory conditions.

## Trial design

The trial is a randomized, single-blind, placebo-controlled study involving a single ascending dose of inhaled R848. The study is not a Clinical Trial of an Investigational Medicinal Product (Non-CTIMP). The study will be conducted in two parts. The first part will involve healthy volunteers (*n* = 24), and the second part will include individuals with mild to moderate asthma (*n* = 6). For each part, participants will be divided into cohorts to receive one of four doses of R848 (0.1, 1, 10, or 100 µg/ml) or placebo. Each cohort will consist of six participants randomly assigned to receive either nebulized R848 or placebo in a 2:1 ratio using REDCap (Research Electronic Data Capture) software. The escalating dose design is intended to identify the highest tolerable dose able to induce an immune response. This approach has been previously used successfully for a therapeutic TLR7 agonist study [11].

## Methods

### Participants

The trial will recruit healthy volunteers and participants with physician-diagnosed mild to moderate asthma, aged 18–60 years (Table 1). Recruitment will be from the Cambridge (UK) area, with additional outreach through volunteer databases, local advertisements, and online platforms. Inclusion criteria for healthy volunteers include the absence of asthma and normal baseline spirometry [FEV1/Forced Vital Capacity (FVC) ratio and FVC > lower limit of normal]. Participants with asthma must not have uncontrolled disease with an Asthma Control Questionnaire (ACQ-5) score of ≤1.5 and be on stable inhaled corticosteroids (ICS) or bronchodilator therapy, with a pre-bronchodilator FEV1 ≥70% predicted. Exclusion criteria include recent respiratory infections, pregnancy, severe obesity (BMI > 40), and a smoking history of more than 5 pack years.

**Table 1.** Inclusion and exclusion criteria

Inclusion criteria			Exclusion criteria
*All participants*	*Participants without asthma*	*Participants with asthma*	*All participants*
Male or Female aged between 18 and 60 years.	No clinical history of asthma.	Physician-diagnosed mild to moderate asthma which is not poorly controlled as evidenced by an Asthma Control Questionnaire (ACQ-5) score of ≤1.5.	No newly prescribed courses of medication including corticosteroids in the four weeks before first study dose other than mild analgesia, vitamins, and supplements.
Willing and able to give informed consent for participation in the study.	Normal baseline spirometry, i.e. FEV1/FVC ratio z-score greater than the lower limit of normal.	They are permitted to be on ICS, long-acting beta agonist, and long-acting muscarinic antagonists.	Participants who have participated in another research study involving an investigational product in the past 12 weeks.
Clinically acceptable laboratory measurements and ECG at enrolment.		Pre-bronchodilator FEV1 ≥70% predicted.	Female participants who are pregnant, lactating or planning pregnancy.
Female participants of child-bearing potential and male participants whose partner is of child-bearing potential must be willing to ensure that they or their partner use effective contraception during the study.		Evidence of bronchial hyperreactivity as evidenced by either (i) Bronchodilator reversibility (increase FEV1 ≥12% and 200 ml); (ii) Positive methacholine challenge (PC20 < 8 mg/ml), or (iii) positive challenge test as per current CUH policy.	Any other significant disease or disorder which, in the opinion of the Investigator, may either put the participants at risk because of participation in the study, or may influence the result of the study, or the participant’s ability to participate in the study.
Ability to expectorate sputum.			Significant extrapulmonary medical conditions.
Optional additional swab for SARS-CoV-2 testing will be collected from participants if required by local and/or national health and safety policies at the time of sampling.			No newly prescribed courses of medication including corticosteroids in the 4 weeks before first study dose other than mild analgesia, vitamins, and supplements.
			Respiratory diseases (other than asthma where specified).
			Upper or lower respiratory tract infection in preceding 14 or 28 days, respectively.
			Smoking tobacco or vaping products in previous 6 months.
			Smoking history of >5 pack years.
			Extreme obesity (BMI >40).

### Interventions

This is a single ascending dose study using nebulized Resiquimod (R848) at four escalating doses (0.1, 1, 10, and 100 µg/ml). Participants will receive a single inhalation of either R848 or saline placebo using a breath-actuated nebulizer (Aeroeclipse II). The dosing for each cohort will be determined based on safety assessments and immune responses from the previous cohort. After receiving the assigned treatment, participants will remain under direct clinical observation for 24 hours and will undergo spirometry and blood sampling at several time points to monitor lung function and immune responses.

### Outcomes

The primary outcome is the change in CXCL10 levels in serum and induced sputum [12] at 24 hours post-inhalation compared with baseline. CXCL10 is a key chemokine in antiviral responses and serves as a biomarker of immune activation [11]. Secondary outcomes include changes in FEV1 at 1, 4-, 8-, 24-, and 48-hours post-inhalation, physiological parameters (temperature, pulse, blood pressure), and inflammatory markers such as C-reactive protein (CRP), eosinophil counts, and lymphocyte counts at 4, 24, and 48 hours.

### Assignment of interventions

Participants will be randomized to receive either inhaled R848 or placebo (saline) in a 2:1 ratio using an electronic randomization system (REDCap). Randomization will be stratified by sex to ensure balanced allocation between treatment arms. The study will be conducted in a single-blind fashion, with participants unaware of treatment allocation. The study team will prepare the solution for nebulization in a separate location to maintain blinding. The nebulized solution will be administered to the participant under direct supervision by trained clinical staff.

## Data collection, management, and analysis

### Data collection

Baseline data, including medical history, physical examination, spirometry, and laboratory tests, will be collected before treatment administration. After inhalation of R848 or placebo, participants will undergo serial spirometry at 1, 4, 8, 24, and 48 hours to assess lung function. Blood samples will be collected at the same intervals for full blood count, CRP, and CXCL10 measurement. Induced sputum samples will be collected 24 hours after exposure to assess immune responses in the lungs. Nasal brushing and nasosorption will be performed at baseline and 24 hours post-exposure to evaluate immune changes in the nasal mucosa.

### Data management

Data will be securely stored in REDCap, and all personal identifiable information will be anonymized following the Data Protection Act 2018. Access to the data will be restricted to authorized personnel involved in the study, and the data will be processed in compliance with Good Clinical Practice standards. The Cambridge Integrated Data Environment (CAM:IDE) System will support digital management of data within the study. It will utilize the REDCap Safe Haven, which is suitable for personally identifiable data and anonymized research data and hosted on the Cambridge University Information Services (UIS) ISO 27001 accredited environment which is also NHS Toolkit Compliant. Biological samples will be predominantly analysed on the Cambridge Biomedical Campus.

### Statistical analysis

The primary outcome will be to assess changes in CXCL10 levels between baseline and post saline or R848 challenge, in serum and sputum samples. We will first perform a statistical test for normality (e.g. Shapiro–Wilks) to determine the most appropriate approach for the comparison between baseline and post-challenge samples. If the data are normally distributed, we will perform *t*-tests. If the data are non-normally distributed, we will perform Wilcoxon signed-rank testing.

Secondary outcomes relating to changes in FEV1, physiological parameters, and inflammatory markers over time will be assessed using repeated measures ANOVA [7]. A significance threshold of *P* < .05 will be used for all statistical tests. Sensitivity analyses will be conducted to account for any missing data points, which will be imputed using the mean of available data from that participant.

## Monitoring and auditing

### Safety monitoring

Participants will be monitored continuously for 24 hours post-inhalation to detect any adverse events (AEs) or serious adverse events (SAEs). Spirometry and physiological parameters (temperature, pulse, and blood pressure) will be recorded at regular intervals. If a participant’s FEV1 decreases by ≥20% from baseline, or if they exhibit significant clinical symptoms, appropriate interventions, including bronchodilator administration, will be provided. All AEs and SAEs will be reported in compliance with regulatory standards, and participants will be followed until any adverse effects resolve or stabilize.

### Auditing

The study will be audited by the University of Cambridge’s risk-based audit program to ensure compliance with the protocol, ethical standards, and regulatory requirements. Auditors will review study documentation, data management procedures, and informed consent records. The study will also be subject to inspection by regulatory bodies as part of the standard oversight for clinical trials conducted at Cambridge University Hospitals NHS Foundation Trust.

## Ethics and dissemination

### Ethical considerations

*In vitro* systems and animal models do not faithfully recapitulate human innate immunity (e.g. mice do not express functional TLR8), and therefore, it is vital to involve human participants, which will also permit the investigation of mechanisms of disease in airway disease such as asthma.

Human live viral challenge studies (such as with influenza) offer a valuable approach to understanding anti-viral immunity but are resource and time intensive, require pre-screening for seropositivity, and may involve quarantine. Furthermore, differences in viral load between groups may confound the comparison of host immunity. R848 challenge offers a complementary approach with administration of identical doses to all volunteers to induce inflammation in a controlled and transient manner.

R848 has previously been safely administered via the oral and topical route at higher doses [13, 14]. Administration of R848 into the respiratory tract has already been established as a suitable and tolerable method for assessing immune responses in the nose [6, 7]. This study will build on these previous studies and develop an approach to deliver the compound and assess immune responses in the lungs.

Participants may experience flu-like symptoms with R848. Risks will be mitigated in several ways: Firstly, nasal R848 administration is well tolerated at different doses (10–100 ug/ml). In the dose finding phase of lower airway challenge with R848, a much lower starting dose of 0.1 ug/ml will be used, and only a single dose given to each volunteer. Secondly, after each dose, there will be careful assessment of observations and symptoms.

The study has undergone extensive Patient and Public Involvement, peer review as well as Research Ethics Committee panel review.

All data will be anonymized and stored securely in accordance with the Data Protection Act 2018. Biological samples will be processed and stored in certified facilities at the Cambridge Biomedical Campus. Participant confidentiality will be maintained throughout the study, and only authorized personnel will have access to identifiable data.

### Dissemination

The results of this study will be disseminated through publications in peer-reviewed journals and presentations at relevant scientific conferences. The results will also be published on clinicaltrials.gov.

## Discussion

The inhaled R848 model offers a unique and controlled method for studying lung-specific immune responses in humans. While previous models have focused on nasal immune responses, the lung presents a distinct environment, particularly in individuals with asthma, where inflammation and structural changes play a critical role in disease progression. The findings from this study will provide insight into the mechanisms of antiviral immunity and inflammation in the lungs and help identify potential therapeutic targets for asthma exacerbations.

The controlled nature of the R848 challenge allows for a reproducible immune stimulus that simulates aspects of viral infections without the risks associated with live pathogens. By comparing responses in healthy individuals and those with asthma, this study will also highlight differences in immune activation that may contribute to disease exacerbations. Furthermore, the model will serve as a valuable platform for testing new anti-inflammatory therapies aimed at reducing the severity of asthma attacks.

## Data Availability

The trial is registered with Clinicaltrials.gov (NCT06488118). The results and data from the trial will be published in an openly accessible manner on this platform at the conclusion of the study.

## Clinical Trial Registration/Status

The trial is currently in the recruitment phase. Data collection is expected to be completed by March 2026, after which the results will be analysed and submitted for publication.

## Permission to reproduce

Graphical abstract created in BioRender. Joseph, J. (2024) BioRender.com/i66f183
